# Relations Between Medical Students’ Motivational Persistence Skills and Their Acceptance of Specific Blended Learning Tools

**DOI:** 10.3390/ejihpe15040045

**Published:** 2025-03-25

**Authors:** Cristina Gena Dascalu, Claudiu Topoliceanu, Magda Ecaterina Antohe

**Affiliations:** 1 Department of Medical Informatics and Biostatistics, Faculty of Medicine, “Grigore T. Popa” University of Medicine and Pharmacy, 16 Universității Street, 700115 Iasi, Romania; 2 Department of Odontology, Periodontology, Fixed Restorations, Faculty of Dental Medicine, “Grigore T. Popa” University of Medicine and Pharmacy, 16 Universității Street, 700115 Iasi, Romania; claudiu.topoliceanu@umfiasi.ro; 3 Department of Implantology, Removable Dentures, Technology, Faculty of Dental Medicine, “Grigore T. Popa” University of Medicine and Pharmacy, 16 Universității Street, 700115 Iasi, Romania; magda.antohe@umfiasi.ro

**Keywords:** blended learning, motivational persistence, psychological evaluation, medical students

## Abstract

The concept of blended education, which refers to the intensive integration of digital resources into the teaching process and its mixed online and on-site delivery, combining as much as possible the advantages of both methods in an optimal way, is becoming increasingly popular among teaching tools. There is no universal recipe for designing a successful blended course; the success of such courses is measured entirely through their degree of acceptance among students, defined by their emotional motivation to learn and the obtained practical results. Our study aimed to evaluate the motivational persistence degree (MPS) of medical students in connection with the students’ acceptance of different didactic tools involved in blended-learning approaches. Materials and Method: We investigated a sample comprising 523 students in Dental Medicine or General Medicine, belonging to all years of study, from four main Universities in Romania; we classified them according to their motivational persistence profile (using k-means data clustering) and we comparatively investigated the main relevant features of students in each cluster—gender, age group, opinions about the general usefulness of multimedia resources in the learning process, and their degree of acceptance of specific types of instructional materials involved in blended learning. Results: We found that the students who mostly enjoy the traditional learning style have average motivational persistence skills; they are perseverant and competitive, but they are not so good at planning their daily tasks. They enjoy external directions, set by teachers. The students who most enjoy PowerPoint presentations and those who enjoy instructional videos show similar behavior, both belonging to the cluster with the highest MPS score. They have the best motivational persistence skills amongst all categories; they are particularly excellent at planning and fulfilling daily tasks, as well as following their goals in the long term. The students who mostly enjoy online documentary sources belong also to a cluster with above average MPS score; they excel in fulfilling daily tasks, but exhibit weaker performance in recalling unachieved goals. These results are similar to those already reported in the literature; the strength of our study is in that it provides much more specific evaluations oriented to the motivational persistence degree, which is highly significant in the case of medical students, because it is a measure of their commitment in fulfilling certain tasks. **Conclusions:** Our results have the potential to highlight reasons for academic success or failure according to a student’ s profile, and will prove helpful in selecting the most appropriate didactic tools.

## 1. Introduction

Modern medical education undergoes a constant process of renewing and updating, because it has to keep pace with technological progress. There is a need to incorporate new tools in order to make it more efficient and to better address the expectations and needs of the current generation of students. This is the so-called ‘Generation Z’ (children born after 1997), also known as iGen, post-millennials, or digital natives. These are children who have been exposed to technology early in life, so that using computers and gadgets has become second nature to them, as has being online and engaging in social networking. The learning style of Generation Z students has features that differentiate it from the learning styles preferred by other generations of students: the most common preferred method is active learning (through discussion and involvement in practical projects) based on sensory stimuli (visual in the form of images and graphics, auditory, and kinesthetic) (Shorey et al., 2021; Szymkowiak et al., 2021). Learning is achieved through observation, practice, and quick and targeted access to the necessary information through the use of technology and online resources (Loveland, 2017), e.g., YouTube for video tutorials, Facebook for discussion with classmates and sharing projects and learning materials, or Google for documentation and searching for scientific references. Students prefer self-paced, individual study with a flexible timetable and multimedia-intensive teaching materials: explanatory or demonstrative videos, PowerPoint presentations with audio support, simulations, collaborative projects, discussion forums, online quizzes, and case studies in the classroom environment (seminar or practical work room).

In this context, the concept of blended education, which refers to the intensive integration of digital resources into the teaching process and its mixed online and on-site delivery, combining as much as possible the advantages of both methods in an optimal way (Graham, 2006; Ruiz et al., 2006), is becoming increasingly popular among teaching tools. The study of this concept and its potential is a topical concern in international research, which has been stimulated all the more in recent years. The COVID-19 pandemic has forced major changes in teaching to be addressed in order to keep university life at a more or less normal pace. In this unique and hopefully unrepeatable context, digital methods and distance learning have emerged as the only viable solutions, which have shown their full usefulness and potential to function as equal partners in the teaching process in the future.

Blended learning combines traditional classroom with modern computer-assisted and multimedia methods, as well as face-to-face and online, and synchronous and asynchronous teaching and learning activities (Bruggeman et al., 2021; Maggio et al., 2018), providing a constant connection between theory and practice and autonomy of learning (Coyne et al., 2018). There are many studies which agree on the significant advantages of this didactic approach: it provides flexibility and mobility, generates interest, and enhances collaboration among students (Kang & Kim, 2021; Ho et al., 2021).

Designing a course in blended format is a complex process, centered around its intended goals, aiming, in fact, to change: the teacher believes that what he is teaching is useful because it can change the lives and evolution of his students for the better, and students are similarly motivated to learn when they believe that what they are learning will enable them to change and evolve, and influence the world around them (Whitaker, 2013). The immediate goals and measurable outcomes of the course must be identified in this interpretation and specified in their natural sequence as a clear map for students, as well as for teachers.

Learning activities usually combine on-site and online formats, with the aim of maximizing the advantages of both methods as much as possible. The digital content has to be designed in a simple and clear manner, according to the standard rules of textbook writing (Adattil, 2018; Hojjati & Muniandy, 2014), and focus should be placed in its capacity to attract and retain the attention of students; it is scientifically proven that the degree of attention with which the audience follows an exposition is at its maximum at the beginning and decreases considerably along the way, because the human brain cannot concentrate on a subject for more than 10 min (Medina, 2009). Practical examples from the teacher’s personal experience may be interspersed between the theory segments to keep the audience interested and emotionally engaged (Brown et al., 2014). Online courses are usually accompanied by on-site, hands-on sessions in which students will have to apply what they have learned (Kyndt et al., 2013). A good practice is to propose projects to be solved through teamwork; in this way, students interact with and support each other and become more responsible, and in addition, can be monitored by the teacher, who individually advises the formed teams.

Assessments are also a highly significant component in any didactic program. In the case of blended courses, the forms of student assessment are more diverse, using both on-site methods, whereby the teacher supervises the exam and directly observes the students’ presentations, and online methods, whereby the students’ work is submitted and checked digitally (Anderson, 2008). On-site assessments are suitable for testing physical skills, human interaction, or the mastery of laboratory procedures; the student performs the tasks required, and the teacher watches him in all aspects and can intervene when necessary. They are also a good solution for oral examinations to assess students’ verbal and non-verbal communication skills and how they interact with their interlocutors. Online assessments offer the advantage of flexibility, saving time and space, and enabling automation and the possibility of repeating them as often as necessary; the most common type of online assessment is the grid test, which is an important tool because it renders the student aware of his/her real level and motivates him/her in the active learning process. Another example is the assessment of assignments and projects on proposed topics, which are designed to demonstrate creativity, critical thinking, analysis and synthesis skills, or knowledge of working methods and tools. Some online assessment activities can be designed so that feedback is obtained not only from the teacher but also from peers through peer assessments.

Finally, another important component is represented by establishing the workflows a student has to go through and creating the proposed activities in order to help the students to understand quickly what they have to do and when (both online and on-site) (Amaral & Shank, 2010). It is recommended to linearly sequence the teaching content, in units identified as Modules, Lessons, or Topics, presented logically and functionally. The course home page should clearly state what is expected of students, i.e., how and when they are to consult the learning content, how they are to communicate with their peers, and how they are to contribute to on-site activities or discussion groups—this will enable students to maintain the teacher’s desired pace from week to week, adapting their learning style to the requirements.

There is no universal recipe for designing a successful blended course; iterative development is the strategy that works best, and involves designing step by step, in small steps. First, design a single unit of a blended course, and if it works, design the other units in the same pattern. If difficulties are encountered during the course of a unit, update the whole structure, applying the changes to all units already designed. However, no matter how well designed, the success of such an approach is measured entirely through its degree of acceptance among students, defined by their emotional motivation to learn and the obtained practical results (their grades at the final examinations).

There are many scientific studies meant to assess students’ motivational beliefs (such as intrinsic and extrinsic goal orientation, self-efficacy, and self-regulated learning strategies (Pintrich, 2000)), because there is a certain positive correlation between them and strong time management skills, information processing, and concentration, which are key factors in academic success (Odontides et al., 2024). Motivation, usually positively correlated with self-efficacy and learning engagement, is essential in medical education, due to the complex, difficult, and time-consuming curriculum (Wu et al., 2020). There are different kinds of motivation, leading to different learning strategies (Yun et al., 2021); for example, goal orientation motivation is associated with elaboration strategy, and acts as a predictor for learning engagement. Pressure to perform, interest, and the importance of the study material are among the main factors driving motivation itself (Schiefele et al., 2003; Zilundu et al., 2022).

Therefore, a holistic approach is required to understand the mechanisms of academic success. Such an approach must combine an understanding of sources of motivation, its assessment, and appropriate learning strategies, in order to stimulate the students’ professional development and to limit possible negative side effects, such as anxiety and depression (Khalil et al., 2017). Proper motivation makes the difference between surface memorization and deep understanding; that is why a good teacher must have insights about his students’ intrinsic and extrinsic motivators, in order to tailor his academic style accordingly. A thorough understanding of students’ motivational drivers leads to enhanced interest, self-efficacy, and long-term retention, improving learning outcomes and the overall academic experience. This is particularly important in the case of medical students; their academic success is mandatory for their future career, because it is directly related to potential patients’ quality of life, and can make sometimes the difference between life and death.

The current literature provides extensive research about the success of blended learning techniques, as well as about students’ motivational persistence skills in relation to their academic success. However, the research aimed at combining these two distinct directions and, moreover, with strict reference to the highly demanding field of medical education, is substantially limited, and usually investigates the issue only through standard methods, without performing in-depth analyses. This is a possible gap we aim to address through our research. We aim to carry out a detailed assessment of medical students’ motivational persistence skills through clustering techniques, which are well-known for their potential in granular analyses, to classify the subjects according to the finest correlations between features, and to relate the resulted clusters, based on motivational profiles, with the students’ preferences for specific tools involved in blended learning programs. Such an approach can reveal valuable insights about the real reasons driving students’ engagement with certain blended learning approaches, leading them to academic success in the long term.

In order to quantify the students’ motivation to learn, we decided to use the Motivational Persistence Scale, proposed by Professor PhD Ticu Constantin, from Al. I. Cuza University’s Psychology Department, located in Iași (Constantin et al., 2011). This scale consists of 42 items and allows quantification of the respondents’ attitudes towards setting long-term goals and following them up, planning and following up on current tasks, and recalling unachieved goals. The Motivational Persistence Scale is recommended to assess a person’s predisposition to persist in tasks or long-term goals that involve ambition, consistency, systematic planning of current activities, focus on accomplishing daily tasks, and frequent updating of unachieved goals.

Our objective aim was to evaluate the motivational persistence degree of medical students by itself, as well as in connection with the students’ acceptance level of different didactic tools involved in blended learning approaches. In order to accomplish this, we classified the students according to their motivational persistence profile, and we comparatively investigated the main relevant features of students in each cluster: gender, age group, opinions about the general usefulness of multimedia resources in the learning process, and their degree of agreement with specific types of instructional materials involved in blended learning.

## 2. Materials and Methods

We recorded the opinions of 523 students in Dental Medicine or General Medicine, spanning all years of study, from four major Universities in Romania (Iași, Craiova, Timisoara, and Cluj-Napoca). The sample’s size was validated according to the calculation made based on a finite population of 63,216 Romanian students enrolled in 2020–2021 within Faculties of Health and Social Assistance, with a confidence level of 95%, and an accepted error of 5%; the study required a minimum of 382 participants.

The selection process was based on voluntary participation via an online survey distributed to students. Respondents who completed the questionnaire selected themselves into the sample based on their availability and distributed the questionnaire link to other potential participants, using snowball sampling to increase the size of the group. Sampling ended by saturation when no more data were recorded on the Google Form.

The sample’s general features are presented in Table 1. Three quarters of the students are female (76.1%); most of them are in years 1 and 2 of study (67.1%) and are aged between 18–20 years (56.8%), with a mean age of 21.47 ± 3.536 years, without statistically significant differences between genders (males have a mean age of 21.93 ± 5.012 years, and females have a mean age of 21.33 ± 2.918 years; *p* = 0.785).

In order to collect the students’ opinions, we used two distinct questionnaires. The first one was a 17-item questionnaire in which the students were invited to express their general opinion on the usefulness of using multimedia resources in the learning process (LMR = agreement to use multimedia resources) and their comparative views regarding specific features of four types of instructional materials used in university education: classic oral presentations (RMT = agreement score for classic oral presentations), PowerPoint presentations (RMP = agreement score for PowerPoint presentations), educational videos (RMF = agreement score for educational videos), and online documentary sources (RMO = agreement score for online documentary sources) (Dascalu et al., 2023). The features surveyed and quantified for each teaching tool were efficacy (utility, value, and completeness), scientific rigor, capacity for synthesis, clarity, and ability to arouse interest.

The second one was the previously mentioned Motivational Persistence Scale questionnaire (Constantin et al., 2011). The questionnaire is made up of 42 items, used to calculate five specific indicators: the students’ skills in setting up ambitious goals (SAG), following their goals in the long term (LTG), planning daily tasks (PDT), fulfilling daily tasks (FDT), and recalling unachieved goals (RUG), as well as the global indicator of motivational persistence (MPS), obtained by summing up the students’ responses to all 42 items.

The questionnaires were adapted and developed starting from models already existent in the scientific literature (Constantin et al., 2011; Alshawish et al., 2021; Yu-Fong Chang et al., 2021; Sezer, 2016; Ibrahim et al., 2021). Their validity was assessed using several methods (expert judgement, item analysis, Kaiser–Meyer–Olkin coefficient, Bartlett test, and factor analysis). Their reliability was estimated by calculating the Cronbach’s alpha coefficients. For the first questionnaire, the Cronbach’s alpha coefficients had the following values: 0.970 (the section regarding classic oral presentations); 0.955 (the section regarding PowerPoint presentations); 0.959 (the section regarding educational videos); and 0.965 (the section regarding online documentary sources). For the second questionnaire, the Cronbach’s alpha coefficients had a global value of 0.924. The separated values for each of the five specific indicators were as follows: 0.791 (the students’ skills in setting up ambitious goals, SAG); 0.762 (the students’ skills in following their goals over the long term, LTG); 0.851 (the students’ skills in planning daily tasks, PDT); 0.842 (the students’ skills in fulfilling daily tasks, FDT); and 0.710 (the students’ skills in recalling unachieved goals, RUG).

On this basis, the questionnaires were considered suitable to be applied in our study.

The questionnaires were presented and explained separately to each subject, along with the research goals. The students were asked to fill out the questionnaires anonymously, online, by specifying their level of agreement with each item on a 5-unit Likert scale (1 = total disagreement, 5 = total agreement). The responses were centralized by calculating overall agreement scores for each of the investigated teaching tools (LMR, RMT, RMP, RMF, and RMO), as well as the scores related to motivational persistence (SAG, LTG, PDT, FDT, RUG, and MPS).

All these calculated scores were analyzed comparatively by gender and age group, as well as in correlation. The results were reported according to the main indications formulated by APA in JARS (Journal Article Reporting Standards), as well as in IBM SPSS’ Documentation Guide, IBM Corporation 2023: the reporting of Cluster Analysis results.

The data from the questionnaire were recorded in a data file in SPSS 29.0 (SPSS Inc., Chicago, IL, USA) for Windows. The answers to each item were characterized by frequency distributions and contingency tables. The numerical variables were characterized through descriptive statistics (mean, standard deviation, range, median, and interquartile range). The comparisons between samples were performed using the Chi-squared test for categorical data and the Mann–Whitney and Kruskal–Wallis tests for quantitative data (after checking the data repartition normality through the Kolmogorov–Smirnov fitting test). We considered a *p* value ≤ 0.05 as statistically significant (*), and a *p* value ≤ 0.01 highly statistically significant (**).

We applied data clustering according to the six scores which measure motivational persistence using the k-means clustering method; we used a maximal number of 40 iterations and a predefined number of two, three, and five clusters. We opted for these particular values because we intended to comparatively investigate additional features of the obtained clusters: their structure in terms of gender (two possible categories), age group (three categories), and the agreement scores for each of the four investigated didactic tools (RMT, RMP, RMF, and RMO), reported on a scale between 1 and 5.

Participation in our study was voluntary. The subjects were informed about the study and the content of the questionnaire, and they agreed to participate and provided informed consent. The study was approved by the Ethical Committee of “Grigore T. Popa” University of Medicine and Pharmacy, Iasi, Romania (decision no. 21/16.11.2020).

## 3. Results

The general data of the resulted scores from the questionnaires, characterized through descriptive statistics, are presented in Table 2.

There are no statistically significant differences between genders concerning agreement on the use of multimedia resources during the learning process; however, the agreement score is almost 10% higher in the case of male students (49.6%) than in the case of female students (39.4%). Female students indicate a significantly higher predisposition for listening to classic oral presentations and PowerPoint presentations, and male students indicate higher enjoyment of online documentary sources (though this observation was not statistically significant). The global indicator of motivational persistence (MPS) is slightly higher for female students than male students, as are the indicators for attitudes toward setting up ambitious goals (SAG), following goals in the long term (LTG), and fulfilling daily tasks (FDT). However, all these differences are not statistically significant. The female students are also significantly better than the male students in planning daily tasks (PDT), while the male students are better, even if not significantly, in recalling unachieved goals (RUG) (Table 3).

There are also no statistically significant differences between age groups concerning the use of multimedia resources during the learning process: the agreement score is almost equal across all three investigated age groups (40.7% of the students aged 18–20 years, 42.6% of the students aged 21–24 years, and 44.9% of the students aged over 25 years). The young students (18–20 years old) exhibit a significant preference for listening to classic oral presentations, while the older students (over 25 years old) exhibit a significant preference for educational videos, as well as online documentary sources. There are no statistically significant differences between age groups regarding the degree of preference for PowerPoint presentations. The global indicator of motivational persistence (MPS) is slightly higher in young students (18–20 years old), although the difference is not statistically significant. The young students (18–20 years old) are also statistically significantly better in setting up ambitious goals (SAG), as well as in following their goals in the long term (LTG). The mature students (over 25 years old) are better (although not statistically significantly) in planning daily tasks (PDT score) and fulfilling them (FDT score), as well as in recalling unachieved goals (RUG) (Table 4).

The first attempt at data clustering according to the six scores relating to motivational persistence, based on two clusters, reached a stable solution in seven iterations. The two identified clusters are well-differentiated, as shown by their final centers, as well as the distance between them, and are relatively balanced in size (Table 5). It is obvious that the global score (MPS) is the most important contributor to defining the clusters, because it is the sum of the other five scores. The score with the next most important contribution is FDT (students’ attitude toward fulfilling daily tasks), followed by PDT (students’ attitude toward planning daily tasks). The students in the second cluster have significantly higher MPS scores than the others; these scores are situated in the upper quartile (Q3: >159), while the scores in the first cluster are situated in the lower quartile (Q1: <130). All the other motivational scores are also significantly higher in the second cluster than in the first one; the differences between them are bigger in the case of FDT, PDT, and LTG scores. In these cases, the scores in the second cluster are again situated in the upper quartile, highlighting the students’ strong skills in planning and fulfilling daily tasks, as well as in following their goals in the long term.

Among the students in the second cluster with strong skills regarding motivational persistence, we find a statistically significant higher percentage of females (80.2%). Half of these students agree or strongly agree with the use of multimedia resources during the learning process (50.0%), compared with only 34.8% of the students in the first cluster; they also have significantly higher scores of agreement regarding all four didactic tools, but they prefer classic oral presentations, followed by educational videos and PowerPoint presentations and, finally, by online documentary sources (Table 6).

The next attempt of data clustering, based on three clusters, reached a stable solution in 21 iterations. The three identified clusters are also well-differentiated, as shown by their final centers; the third cluster is the smallest, followed by the first one, and the second cluster is the biggest. The biggest difference is between the first and the first cluster, with the second cluster between them (Table 7). The global score (MPS) again exhibits the most important contribution in defining the clusters, followed by FDT (students’ attitude toward fulfilling daily tasks). This time, however, the other scores make significantly smaller contributions to defining the three clusters. The next most influential is SAG (students’ attitude toward setting up ambitious goals). The first cluster contains the students with the lowest MPS score, smaller than the lower quartile (Q1: <130); the second cluster contains the students with average MPS scores, close to the global median value (Q2: 143); and the third cluster contains the students with the highest MPS scores, bigger than the upper quartile (Q3: >159). All the other motivational scores follow the same trend, showing that the students in the third cluster have strong skills regarding them, the students in the second cluster have average skills, and the students in the first cluster have the weakest skills.

There are no statistically significant differences between gender and age group across the three identified clusters, although the percentage of female students is slightly higher in the third cluster (80.3%), as well as the percentage of young students (18–20 years old, 61.3%). The highest percentage of students who agree with the use of multimedia resources during the learning process is again found within the third cluster (52.1%), while the highest percentage of students who do not agree with the use of multimedia resources during the learning process is found within the first cluster (25.3%). The students in the second cluster, with average skills regarding motivational persistence, are neutral towards (38.9%) or in agreement with (24.6%) the use of multimedia resources during the learning process. Classic oral presentations are most popular amongst the students in the second cluster, with average motivational persistence skills; the other three investigated didactic tools have the highest scores among the students in the third cluster, with the strongest motivational persistence skills (Table 8).

The last attempt at data clustering, based on five clusters, reached a stable solution in 20 iterations. The five identified clusters are also well-differentiated, as shown by their final centers; the first and second clusters are smallest, and the other three are bigger. The biggest difference is between the first and the 2nd cluster, followed by the difference between the first and the fifth clusters; other different clusters are the second and the third, and the closest clusters are the fourth and the fifth, as well as the third and the fourth and the second and the fifth (Table 9). The global score (MPS) makes the most important contribution in defining the clusters, followed by the FDT (students’ attitude toward fulfilling daily tasks) and PDT (students’ attitude toward planning daily tasks). The first cluster contains the students with the highest values for MPS score, situated in the upper quartile (Q3: >159); these students have also high values for all the other motivational scores, situated in the corresponding upper quartiles, therefore showing strong skills regarding them. The next cluster is the third one; the MPS score in its center is also situated in the upper quartile, showing a high level of motivational persistence, but this time, all the other five composing scores are slightly lower than the corresponding upper quartiles, so the corresponding skills are slightly attenuated. The next cluster is the fourth one, with an MPS score in its center situated close to the global median value, as well as all the other five composing scores; the students in this cluster, which is actually the biggest in size, have an average level of motivational persistence. The last clusters are the fifth and the second, in this order; in both of them, the MPS scores belong to the lowest quartile (Q1: <130). The students in the fifth cluster exhibit average skills in setting up ambitious goals (SAG), planning daily tasks (PDT), and recalling unachieved goals (RUG), i.e., the corresponding scores are bigger than the lowest quartiles, while the students in the second cluster have the weakest skills related to all five investigated indicators.

Again, there are no statistically significant differences between gender and age groups in the structures of the five identified clusters, even if the highest percentage of females (86.2%) is found within the first cluster, with the strongest motivational persistence skills, while the highest percentage of males (30.4%) is found within the last cluster, with the weakest motivational persistence skills. The highest percentage of young students (18–20 years old, 63.8%) is also found within the first cluster. The students with the strongest motivational persistence skills agree in more than half the cases (56.9%) with the use of multimedia resources during the learning process; the students with average skills agree with this learning style in 42.4% of cases, and the students with the weakest skills agree in 41.3% of cases. Classic oral presentations are mostly preferred by the students in the third cluster, with average motivational persistence skills, and least preferred by the students with the weakest motivational persistence skills. PowerPoint presentations are mostly preferred by the students in the first cluster, with the strongest motivational persistence skills, and least preferred again by the students in the last cluster, with the weakest skills. The educational videos are also mostly preferred by the students with the strongest motivational persistence skills, and least preferred by the students with weak and the weakest skills (last two identified clusters). The online documentary sources are mostly preferred by the students with strong motivational persistence skills, but not the strongest (the second cluster), and least preferred by the students in the last cluster, with the weakest skills. All these differences are again statistically significant (Table 10).

## 4. Discussion

There is extensive research surrounding the relationship between the psychological profile of medical students and their academic performance. Our study also contributes to this research direction.

The scientific literature reveals that the academic success of medical students is often related to the presence of certain personality traits; the most well-documented features in this regard are conscientiousness (highly conscientious students tend to perform better academically (Dudley et al., 2006)), resilience and stress management (resilient students, resistant to being overwhelmed by stress, also achieve better academic results (Tempski et al., 2012)), emotional intelligence (students able to recognize and to manage their own emotions as well as those of others have better interpersonal skills, crucial for clinical settings, and are also better at coping with stress and maintaining motivation (Arora et al., 2010)), intrinsic motivation (medical students driven by a genuine interest in learning and self-improvement have usually better academic results than those driven by rewards, grades, or recognition (Kusurkar et al., 2011)), self-regulation and time management skills (good students are self-disciplined and able to set goals, to monitor their progress, and to adapt their learning and thinking styles—visual, auditory, kinesthetic/analytical, or holistic—to their cognitive strengths, and to adjust their learning strategies if necessary (Cook et al., 2011)), and social support (students with positive support from peers, family, or mentors perform better than those without).

Other personality traits have also been investigated, but with inconsistent results as to their relationship with academic success (Lievens et al., 2002); these include openness to experience (students curious about obtaining new knowledge are better at critical thinking and more well-prepared for problem-based learning, but this is not necessarily related with obtaining good grades), extraversion (extraverted students have good communication and interpersonal skills, helpful in clinical settings, but they can have serious difficulties during intensive study time, required especially in pre-clinical years), agreeableness (this trait aids students in team-based learning environments, but it is not necessarily related with individual study and performance), and perfectionism (striving for excellence can drive students to work hard and achieve high grades, but can also cause stress, procrastination, and burnout, especially when students cannot accept failure (Gracia & Molero, 2013)). Finally, there are also personality traits with demonstrated negative influences on academic performance; this includes neuroticism (emotional instability, which usually leads to high levels of stress and poor concentration and time management), as well as different mental health issues (anxiety, depression, or burnout; medical students are particularly prone to these issues due to the high stress load and competitive nature of their studies).

The success of blended learning approaches depends also on the students’ personality traits, and especially on conscientiousness, resilience, self-regulation, emotional intelligence, and intrinsic motivation (the same features required for the academic success of medical students), combined additionally with openness to experience, adaptability, autonomy, and adaptive perfectionism (Broadbent & Poon, 2015; Alvi & Gillies, 2021; Molnar, 2019).

The scientific literature has also identified the main psychological features of students who enjoy particular didactic tools which belong to the blended learning approach, as follows:

Students who enjoy the traditional learning style, based on classic oral presentations, enjoy teacher-led instruction, focused on lectures, textbooks, and written exams, showing therefore a clear preference for hierarchy and authority within the classroom (Chetty et al., 2019; Gargallo López et al., 2013). From a psychological perspective, these students prefer predictable environments and routine, as well as clearly outlined tasks and detailed instructions; they value discipline and tend to follow rules, perceiving the teachers as authority figures and a primary source of knowledge and guidance. They often exhibit high conscientiousness, materialized in strong work ethic, responsibility, and attention to details; they also tend to be motivated by grades and any form of external validation, with completing assignments on time and doing well on exams being of importance to them (Khalilzadeh & Khodi, 2021). In some cases, they can be perfectionists, doing their best to exceed set expectations. They are linear thinkers, enjoying concrete information, memorization, rote learning, and the exact sciences, based on clearly right or wrong answers (Cimermanová, 2018); on the other hand, they may be rather resistant to adopting new technologies or pedagogical innovations, being less comfortable with experimentation or creative approaches to learning (Poropat, 2009). They also enjoy working independently rather than in groups, being focused on their individual performance and success. They tend to be competitive and self-confident; it is important to them to feel that they can control their outcomes by following a set plan, because they believe in their ability to perform well when given clear instructions, and in a stable and consistent environment.

According to our study, the students who primarily enjoy such a traditional learning style have average motivational persistence skills. The MPS global score of the cluster’s center (142.78) is close to the global average of this score (144.19 ± 20.231), as well as to its median (143.00). The SAG score (34.66) is almost equal with the global average (34.36 ± 5.047) and to the median (34.00), as well as the RUG score (24.73, compared with its global average 24.89 ± 4.673 and median 24.00), showing average skills in setting up ambitious goals and recalling unachieved goals. The PDT and FDT scores are slightly smaller than the global average and median (PDT: 27.68 compared with 28.71 ± 6.607/28.00, FDT: 26.67 compared with 27.29 ± 5.214/27.00), showing weaker skills in planning and fulfilling daily tasks, while the LTG score is slightly superior to the global average and median (29.04 compared with 28.94 ± 4.506/29.00), showing good skills in following goals in the long term. These students are therefore perseverant and competitive, but, since they do not particularly enjoy planning their daily tasks, it follows that they enjoy external directions set by teachers. This finding is similar to those reported in the literature.

Students who enjoy learning through PowerPoint presentations also have specific psychological traits, aligned with the structured and visual nature of this format. They are active-visual learners who tend to prefer visual and creative forms of information, and are more likely to understand and retain knowledge when it is presented through images, diagrams, and charts (Mayer, 2009; Bartsch & Cobern, 2003). Sometimes, they need more stimulation to stay attentive in class. They appreciate clarity and brevity and love sequential, organized, and methodical learning, based on concepts that are built upon each other in a structured way (Onivehu & Ohawuiro, 2018). This sort of consistent format helps them to feel in control with their learning, especially when the flow of information is predictable and well-structured. They often have strong short-term memory skills, facilitating them to retain and process the information presented in segmented form (like the concise bullet points and key concepts typically found on PowerPoint slides) (Onivehu & Ohawuiro, 2018; Kalyuga, 2011). They also enjoy well-structured learning content and its division into specific tasks or objectives. It is helpful for them to set targets and to focus on them. They value efficiency and information delivery in a concise manner, without long explanations or discussions (Apperson et al., 2008).

They may enjoy the external guidance provided by a teacher, but they equally search for opportunities for interaction, such as class discussions and question-answer sessions. They are mainly individual learners, since PowerPoint is a format typically used for content delivery rather than collaborative learning. They are comfortable with technology and multimedia, appreciating digital tools and resources that enhance learning, as well as the multi-sensory approach to education, based on auditory and visual stimuli aiming to reinforce learning (Ozaslan & Maden, 2013).

Another investigated didactic tool was represented by educational videos. Students who enjoy using this tool also have specific psychological features. They usually belong to the category of visual or auditory learners (Mayer, 2009; Berk, 2009), which means that they learn best when they receive multimodal information, through multiple sensory channels: visual demonstrations supported by explanations. Therefore, they prefer dynamic and interactive content over static or text-based materials, as well as active engagement through hands-on sessions, often having strong visual memory and multitasking skills (Giannakos, 2013) and low tolerance for monotony. They are also self-directed learners who enjoy controlling the pace and timing of their learning (Kay, 2012), as well as active learners: they interact with the video content by taking notes and asking themselves questions as they watch. They value the real-world application of knowledge, because videos present case studies, demonstrations, and practical examples that connect abstract concepts to real-life situations (Park et al., 2011); they tend to be global learners, focused on understanding the “big picture” first and only thereafter the finer concepts and details. Similar to the students who enjoy PowerPoint presentations, those who enjoy educational videos also appreciate clear learning content that is well-structured and divided into specific tasks and objectives (Guo et al., 2014), as well as external guidance through narration; they may struggle to focus on long lectures or textbook readings, having shorter attention spans. They have a high degree of openness to experience, being willing to explore new and creative ways of learning; they are also open to technology, being comfortable with digital tools and online platforms that host didactic videos.

In our study, the students who enjoy PowerPoint presentations the most and those who enjoy instructional videos showed similar behavior, with both of them belonging to the cluster with the highest MPS score (179.79 in the cluster’s center, compared with the global reported average of 144.19 ± 20.231, median of 143.00, and third quartile (Q3) of 159.00). These students have the best motivational persistence skills amongst all categories. All five component scores are high and superior to the average and the third quartile (Q3) reported globally (SAG: 40.50 compared with 34.36 ± 5.047/38.00; LTG: 35.00 compared with 28.94 ± 4.506/32.00; PDT: 39.19 compared with 28.71 ± 6.607/33.00; FDT: 35.29 compared with 27.29 ± 5.214/31.00; and RUG: 29.81 compared with 24.89 ± 4.673/28.00). They therefore have the best skills in setting ambitious goals and following them in the long term, planning and fulfilling daily tasks, and recalling unachieved goals; they are particularly excellent in planning and fulfilling daily tasks, as well as in following their goals in the long term. Our findings confirm therefore that such students are self-directed learners, able to set and to control their learning pace. They value the efficacy of learning materials, and they are conscientious, responsible, and pragmatic, aware of their goals in the short and long term, and assuming these goals to be in their reach.

The last category of investigated students was represented by those who enjoy learning through online documentary sources. These students are naturally curious and have a natural inclination to explore and learn more about a topic, being driven by a strong internal desire to understand and solve problems (Mayer, 2005; Sato et al., 2022). They are introverted, preferring to process information on their own before seeking out external input. They are self-starters, flexible, and analytical; they enjoy breaking down complex concepts and understanding the logical flow of systems, preferring to seek answers independently, slowly and methodically, rather than relying on others for explanations. They tend to be persistent problem solvers; they are not easily discouraged by challenges and are willing to invest the time to dig through detailed information to find solutions, also being capable of shifting their thinking to accommodate new information (Mustafa et al., 2022). They are detail-oriented and patient, searching for comprehensive and in-depth information rather than superficial explanations, and they enjoy the clarity and thoroughness provided by structured, step-by-step reference manuals. They are also good at figuring things out despite gaps, and are able to deal with uncertainty or incomplete information. They are comfortable working independently rather than engaging in collaborative learning environments, because they prefer to figure things out on their own, without immediate feedback or social interaction from classrooms or working groups (Dabbagh & Kitsantas, 2012). They are very comfortable with technology, finding the digital medium engaging and intuitive for their learning process (Mustafa et al., 2022); they have natural aptitudes that enable them to independently learn how to use new tools and to adapt quickly to different technical environments (Yu et al., 2022). They are often goal-oriented learners, with good self-regulation and time management skills (Sato et al., 2022), allowing them to work methodically toward a specific goal and to dedicate the appropriate amount of time to it.

In our study, the students who mostly enjoy online documentary sources belonged also to a cluster with an above average MPS score (159.32 in the cluster’s center, compared with the global average of 144.19 ± 20.231, median of 143.00, and third quartile (Q3) of 159.00), indicating that they also have superior motivational persistence skills. All five component scores follow the same trend: they are superior to the global average and median, being close to, but slightly smaller than, the 3rd quartile (Q3) (SAG: 37.50 compared with 34.36 ± 5.047/34.00/38.00; LTG: 31.46 compared with 28.94 ± 4.506/29.00/32.00; PDT: 32.56 compared with 28.71 ± 6.607/28.00/33.00; FDT: 30.99 compared with 27.29 ± 5.214/27.00/31.00; and RUG: 26.80 compared with 24.89 ± 4.673/24.00/28.00). These students therefore have superior skills in setting up ambitious goals and following them in the long term, planning and fulfilling daily tasks, and recalling unachieved goals. Their performance levels are similar in terms of setting up ambitious goals, following them in the long term, and planning daily tasks; they even excel in fulfilling daily tasks, but are slightly weaker in terms of recalling unachieved goals. These findings confirm the previously reported results, according to which they are independent learners with high self-regulation skills, as well as persistent problem solvers.

Our study generally confirms the results already reported in the literature, bringing also new insights into the psychological profiles of students who prefer certain didactic tools over others. We highlighted a clear differentiation between students in terms of their motivational persistence skills. Regardless of the predefined number of clusters that we chose, three groups stood out most clearly: students with high values for these skills, those located at medium levels, and those with weak skills. Girls belong mainly to the first category, as do the youngest students aged between 18 and 20; students in this category systematically agree with the use of multimedia resources in the learning process. As students’ motivational persistence skills decrease, their appetite for integrating multimedia resources into the learning process also decreases statistically significantly. Regarding the preference for certain teaching tools, an interesting fact is that students’ preferences are ranked similarly, regardless of their level of motivational persistence: in first place are traditional presentations, followed by instructional videos, then by PowerPoint presentations, while online documentary sources are in last place. 

However, in-depth analysis of the clustering results offers specific, new insights: students with high motivational persistence skills like instructional videos and PowerPoint presentations more than others, those with intermediate skills like traditional presentations the most, or, when their cumulative score of MPS skills is higher, they like online documentary sources. Students with a low overall MPS score and low potential in following goals in the long term (LTG) and fulfilling daily tasks (FDT) like instructional videos as much as PowerPoint presentations, and those with low values for all motivational persistence scores like PowerPoint presentations as little as online documentary sources.

All this is new, targeted, and accurate information which explains students’ preferences for certain teaching tools in direct relation to their psychological profile. Clustering techniques provide the capacity to perform in-depth analysis in large datasets and to highlight such fine internal connections. The quality of knowledge provided is the strength of our study, allowing us to better understand the reasons that some students prefer certain didactic tools over others in relation to their abilities to manage their daily and long-term goals, which are directly connected with academic success. This is particularly important in the case of medical students, in order to develop didactic tools better tailored to their real needs and expectancies, which play an essential role in their professional training.

The main limitation of our study is its non-interventional design. We intend to continue our research in a more interventional manner, by setting up an adaptive learning model based on the recorded results of the motivational persistence survey. Such a model can eventually involve machine learning algorithms in order to dynamically adjust instructional tools based on students’ motivational profiles, therefore providing a fully customized learning experience. This is an innovative approach, suitable for the development of modern blended medical learning programs. The advantages of such approaches are undeniable: the teacher can better understand how students with different motivational persistence profiles engage with various learning tools, becoming able to make informed decisions in optimizing their teaching strategy by selecting the most appropriate didactic tools according to the students’ profile.

## 5. Conclusions

Nowadays, blended learning is the next step in education improvement based on technological advances. It plays a transformative role in medical education by combining traditional in-person instruction with online or digital resources, providing flexibility, engagement, and efficiency, and enhancing the overall learning experience. In order to successfully apply these tools and to maximize their advantages, the teacher must select them according to his students’ native abilities to set daily and long-term goals and to engage in reaching them; this is the best way to lead students to academic success. It is a complex task, involving psychological evaluations and, sometimes, advanced data analysis skills. Our study highlights the importance of considering motivational persistence when selecting blended learning tools for medical students. By integrating complex data analysis techniques (i.e., data clustering), we acquired a deeper understanding of how students engage with different instructional strategies. Such insights can guide educators in designing more personalized, effective curricula that align with students’ intrinsic motivational tendencies, ultimately enhancing learning outcomes and academic performance.

## Figures and Tables

**Table 1:** General features of the study group.

		*n*	%
Gender	male	125	23.9
	female	398	76.1
Age group	18–20 years	297	56.8
	21–24 years	157	30.0
	over 25 years	69	13.2
Year of study	1	228	43.6
	2	123	23.5
	3	44	8.4
	4	2	0.4
	5	44	8.4
	6	82	15.7
Faculty	General Medicine	190	36.3
	Dental Medicine	262	50.1
	Dental Technique	71	13.6
University	UMF “Grigore T. Popa” Iași	328	62.7
	UMF Craiova	108	20.7
	UMF ”Victor Babeș” Timișoara	80	15.3
	UMF ”Iuliu Hațieganu” Cluj Napoca	7	1.3
Total		523	100.0

**Table 2:** The students’ agreement level with specific didactic tools involved in blended learning and their motivational persistence level: descriptive statistics.

	N (%)		
LMR:			
1	20 (3.8)		
2	78 (14.9)		
3	206 (39.4)		
4	111 (21.2)		
5	108 (20.7)		
	m ± SD	min ÷ max	Q1/Median/Q3
Agreement scores for specific didactic tools:			
RMT	4.03 ± 0.905	1.00 ÷ 5.00	3.56/4.19/4.81
RMP	3.43 ± 0.835	1.00 ÷ 5.00	2.94/3.50/4.00
RMF	3.59 ± 0.885	1.00 ÷ 5.00	3.00/3.81/4.25
RMO	3.30 ± 0.939	1.00 ÷ 5.00	2.69/3.31/4.00
Motivational persistence scores:			
MPS	144.19 ± 20.231	75 ÷ 198	130/143/159
SAG	34.36 ± 5.047	17 ÷ 45	30/34/38
LTG	28.94 ± 4.506	15 ÷ 40	26/29/32
PDT	28.71 ± 6.607	10 ÷ 45	24/28/33
FDT	27.29 ± 5.214	10 ÷ 40	24/27/31
RUG	24.89 ± 4.673	12 ÷ 40	22/24/28

**LEGEND**: **LMR** = agreement with the use of multimedia resources; **RMT** = agreement score for classic oral presentations; **RMP** = agreement score for PowerPoint presentations; **RMF** = agreement score for educational videos; **RMO** = agreement score for online documentary sources; **MPS** = global indicator of motivational persistence; **SAG** = attitude toward setting up ambitious goals; **LTG** = attitude toward following goals in the long term; **PDT** = attitude toward planning daily tasks; **FDT** = attitude toward fulfilling daily tasks; **RUG** = attitude toward recalling unachieved goals.

**Table 3:** The students’ agreement level surrounding specific didactic tools involved in blended learning and their motivational persistence level: comparative study on genders.

	Gender		*p*-Value
	Male (n = 125)	Female (n = 398)	
	N (%)	N (%)	
LMR:			0.378 †
1	4 (3.2)	16 (4.0)	
2	15 (12.0)	63 (15.8)	
3	44 (35.2)	162 (40.7)	
4	32 (25.6)	79 (19.8)	
5	30 (24.0)	78 (19.6)	
	m ± SD min ÷ max/median	m ± SD min ÷ max/median	
Agreement scores for specific didactic tools:			
RMT	3.83 ± 1.037 1.06 ÷ 5.00/4.00	4.09 ± 0.852 1.00 ÷ 5.00/4.25	0.017 ‡*
RMP	3.28 ± 0.878 1.00 ÷ 5.00/3.31	3.48 ± 0.817 1.19 ÷ 5.00/3.50	0.022 ‡*
RMF	3.47 ± 0.987 1.00 ÷ 5.00/3.63	3.63 ± 0.848 1.00 ÷ 5.00/3.81	0.099 ‡
RMO	3.36 ± 0.996 1.00 ÷ 5.00/3.31	3.29 ± 0.921 1.00 ÷ 5.00/3.31	0.566 ‡
Motivational persistence scores:			
MPS	141.52 ± 19.958 98 ÷ 195/139	145.03 ± 20.270 75 ÷ 198/144	0.072 ‡
SAG	33.76 ± 5.560 19 ÷ 45/34	34.55 ± 4.867 17 ÷ 45/35	0.092 ‡
LTG	28.69 ± 4.321 17 ÷ 38/29	29.02 ± 4.565 15 ÷ 40/29	0.514 ‡
PDT	26.91 ± 6.365 11 ÷ 45/27	29.27 ± 6.589 10 ÷ 44/29	<0.001 ‡**
FDT	26.98 ± 5.200 13 ÷ 40/27	27.39 ± 5.221 10 ÷ 40/27	0.411 ‡
RUG	25.18 ± 4.842 12 ÷ 39/25	24.79 ± 4.621 13 ÷ 40/24	0.416 ‡

† Pearson Chi-squared test; ‡ Mann–Whitney U test; * *p* < 0.05 statistically significant; ** *p* < 0.01 highly statistically significant. **LEGEND**: **LMR** = agreement with the use of multimedia resources; **RMT** = agreement score for classic oral presentations; **RMP** = agreement score for PowerPoint presentations; **RMF** = agreement score for educational videos; **RMO** = agreement score for online documentary sources; **MPS** = global indicator of motivational persistence; **SAG** = attitude toward setting up ambitious goals; **LTG** = attitude toward following goals in the long term; **PDT** = attitude toward planning daily tasks; **FDT** = attitude toward fulfilling daily tasks; **RUG** = attitude toward recalling unachieved goals.

**Table 4:** The students’ agreement level on specific didactic tools involved in blended learning and their motivational persistence level: comparative study on age groups.

	Age Group			*p*-Value
	18–20 years (n = 297)	21–24 years (n = 157)	Over 25 years (n = 69)	
	N (%)	N (%)	N (%)	
LMR:				0.730 †
1	14 (4.7)	4 (2.5)	2 (2.9)	
2	49 (16.5)	22 (14.0)	7 (10.1)	
3	113 (38.0)	64 (40.8)	29 (42.0)	
4	57 (19.2)	36 (22.9)	18 (26.1)	
5	64 (21.5)	31 (19.7)	13 (18.8)	
	m ± SD min ÷ max/median	m ± SD min ÷ max/median	m ± SD min ÷ max/median	
Agreement scores for specific didactic tools:				
RMT	4.16 ± 0.840 1.00 ÷ 5.00/4.38	3.84 ± 0.973 1.00 ÷ 5.00/4.00	3.91 ± 0.940 1.06 ÷ 5.00/4.06	0.001 ‡**
RMP	3.45 ± 0.828 1.06 ÷ 5.00/3.44	3.35 ± 0.891 1.00 ÷ 5.00/3.44	3.56 ± 0.719 1.19 ÷ 5.00/3.63	0.150 ‡
RMF	3.49 ± 0.892 1.00 ÷ 5.00/3.63	3.66 ± 0.867 1.00 ÷ 5.00/3.88	3.89 ± 0.818 1.06 ÷ 5.00/3.94	0.001 ‡**
RMO	3.16 ± 0.956 1.00 ÷ 5.00/3.13	3.37 ± 0.888 1.25 ÷ 5.00/3.38	3.73 ± 0.833 1.06 ÷ 5.00/3.81	<0.001 ‡**
Motivational persistence scores:				
MPS	145.01 ± 20.283 75 ÷ 196/144	142.42 ± 19.445 90 ÷ 195/141	144.68 ± 21.760 105 ÷ 198/140	0.368 ‡
SAG	34.89 ± 5.154 17 ÷ 45/35	33.73 ± 4.748 22 ÷ 45/33	33.54 ± 5.034 25 ÷ 45/33	0.009 ‡**
LTG	29.33 ± 4.706 15 ÷ 39/30	28.29 ± 4.064 16 ÷ 40/29	28.75 ± 4.460 22 ÷ 40/28	0.022 ‡*
PDT	28.68 ± 6.608 10 ÷ 44/28	28.46 ± 6.543 11 ÷ 44/29	29.38 ± 6.780 15 ÷ 45/28	0.797 ‡
FDT	27.38 ± 5.290 10 ÷ 39/27	26.98 ± 4.776 15 ÷ 40/27	27.59 ± 5.847 14 ÷ 40/27	0.659 ‡
RUG	24.72 ± 4.805 12 ÷ 39/24	24.96 ± 4.631 15 ÷ 38/24	25.42 ± 4.188 17 ÷ 40/25	0.614 ‡

† Pearson Chi-squared test; ‡ Kruskal–Wallis H test; * *p* < 0.05 statistically significant; ** *p* < 0.01 highly statistically significant. **LEGEND**: **LMR** = agreement with the use of multimedia resources; **RMT** = agreement score for classic oral presentations; **RMP** = agreement score for PowerPoint presentations; **RMF** = agreement score for educational videos; **RMO** = agreement score for online documentary sources; **MPS** = global indicator of motivational persistence; **SAG** = attitude toward setting up ambitious goals; **LTG** = attitude toward following goals in the long term; **PDT** = attitude toward planning daily tasks; **FDT** = attitude toward fulfilling daily tasks; **RUG** = attitude toward recalling unachieved goals.

**Table 5:** Main features of the two-clusters solution.

	Final Cluster Centers—The Typical Case for Each Cluster		ANOVA		Distances Between Final Cluster Centers
	Cluster		F	*p*-Value	
	1 (n = 281)	2 (n = 242)			
	Mean ± SD	Mean ± SD			
MPS	128.91 ± 11.319	161.93 ± 12.235	1026.224	<0.001 **	1–2: 36.339
SAG	31.41 ± 4.143	37.80 ± 3.631	346.698	<0.001 **	
LTG	26.18 ± 3.560	32.15 ± 3.165	404.955	<0.001 **	
PDT	24.56 ± 4.485	33.52 ± 5.278	440.704	<0.001 **	
FDT	23.83 ± 3.632	31.31 ± 3.647	550.494	<0.001 **	
RUG	22.94 ± 3.893	27.14 ± 4.492	131.341	<0.001 **	

** *p* < 0.01 highly statistically significant. **LEGEND**: **MPS** = global indicator of motivational persistence; **SAG** = attitude toward setting up ambitious goals; **LTG** = attitude toward following goals in the long term; **PDT** = attitude toward planning daily tasks; **FDT** = attitude toward fulfilling daily tasks; **RUG** = attitude toward recalling unachieved goals.

**Table 6:** Additional features of the two-clusters solution.

		Cluster				*p*-Value
		1 (n = 281)		2 (n = 242)		
		N	%	N	%	
Gender	male	77	27.4%	48	19.8%	0.043 †*
	female	204	72.6%	194	80.2%	
Age group	18–20 years	156	55.5%	141	58.3%	0.795 †
	21–24 years	86	30.6%	71	29.3%	
	over 25 years	39	13.9%	30	12.4%	
LMR	1	13	4.6%	7	2.9%	<0.001 †**
	2	56	19.9%	22	9.1%	
	3	114	40.6%	92	38.0%	
	4	58	20.6%	53	21.9%	
	5	40	14.2%	68	28.1%	
		m ± SD min ÷ max/median		m ± SD min ÷ max/median		
RMT		3.97 ± 0.873 1.06 ÷ 5.00/4.06		4.10 ± 0.939 1.00 ÷ 5.00/4.25		0.021 ‡*
RMP		3.28 ± 0.831 1.06 ÷ 5.00/3.31		3.60 ± 0.808 1.00 ÷ 5.00/3.66		<0.001 ‡**
RMF		3.44 ± 0.882 1.00 ÷ 5.00/3.63		3.77 ± 0.857 1.00 ÷ 5.00/3.94		<0.001 ‡**
RMO		3.14 ± 0.907 1.00 ÷ 5.00/3.13		3.49 ± 0.940 1.00 ÷ 5.00/3.59		<0.001 ‡**

† Pearson Chi-squared test; ‡ Mann–Whitney U test; * *p* < 0.05 statistically significant; ** *p* < 0.01 highly statistically significant. **LEGEND**: **LMR** = agreement with the use of multimedia resources; **RMT** = agreement score for classic oral presentations; **RMP** = agreement score for PowerPoint presentations; **RMF** = agreement score for educational videos; **RMO** = agreement score for online documentary sources.

**Table 7:** Main features of the three-clusters solution.

	Final Cluster Centers—The Typical Case for Each Cluster			ANOVA		Distances between Final Cluster Centers
	Cluster			F	*p*-Value	
	1 (n = 170)	2 (n = 211)	3 (n = 142)			
	Mean ± SD	Mean ± SD	Mean ± SD			
MPS	122.08 ± 9.330	144.97 ± 6.745	169.49 ± 10.408	1144.971	<0.001 **	1–2: 25.137
SAG	29.76 ± 3.667	34.77 ± 3.612	39.27 ± 2.939	294.756	<0.001 **	1–3: 52.127
LTG	24.88 ± 3.227	29.31 ± 3.304	33.25 ± 2.764	277.242	<0.001 **	2–3: 27.018
PDT	23.19 ± 4.212	28.53 ± 4.592	35.56 ± 4.991	281.591	<0.001 **	
FDT	22.35 ± 3.406	27.43 ± 3.142	33.01 ± 3.105	424.181	<0.001 **	
RUG	21.90 ± 3.653	24.93 ± 3.673	28.39 ± 4.649	104.305	<0.001 **	

** *p* < 0.01 highly statistically significant. **LEGEND**: **MPS** = global indicator of motivational persistence; **SAG** = attitude toward setting up ambitious goals; **LTG** = attitude toward following goals in the long term; **PDT** = attitude toward planning daily tasks; **FDT** = attitude toward fulfilling daily tasks; **RUG** = attitude toward recalling unachieved goals.

**Table 8:** Additional features of the three-clusters solution.

		Cluster						*p*-Value
		1 (n = 170)		2 (n = 211)		3 (n = 142)		
		N	%	N	%	N	%	
Gender	male	43	25.3%	54	25.6%	28	19.7%	0.391 †
	female	127	74.7%	157	74.4%	114	80.3%	
Age group	18–20 years	90	52.9%	120	56.9%	87	61.3%	0.623 †
	21–24 years	57	33.5%	61	28.9%	39	27.5%	
	over 25 years	23	13.5%	30	14.2%	16	11.3%	
LMR	1	6	3.5%	8	3.8%	6	4.2%	0.004 †*
	2	37	21.8%	30	14.2%	11	7.7%	
	3	73	42.9%	82	38.9%	51	35.9%	
	4	27	15.9%	52	24.6%	32	22.5%	
	5	27	15.9%	39	18.5%	42	29.6%	
		m ± SD min ÷ max/median		m ± SD min ÷ max/median		m ± SD min ÷ max/median		
RMT		3.88 ± 0.876 1.50 ÷ 5.00/4.00		4.12 ± 0.828 1.06 ÷ 5.00/4.31		4.08 ± 1.026 1.00 ÷ 5.00/4.25		0.004 ‡**
RMP		3.23 ± 0.797 1.31 ÷ 5.00/3.19		3.42 ± 0.863 1.00 ÷ 5.00/3.50		3.69 ± 0.770 1.31 ÷ 5.00/3.81		<0.001 ‡**
RMF		3.33 ± 0.903 1.00 ÷ 5.00/3.47		3.64 ± 0.828 1.00 ÷ 5.00/3.81		3.84 ± 0.865 1.06 ÷ 5.00/4.00		<0.001 ‡**
RMO		3.12 ± 0.920 1.00 ÷ 5.00/3.09		3.31 ± 0.887 1.00 ÷ 5.00/3.25		3.50 ± 0.999 1.00 ÷ 5.00/3.63		<0.001 ‡**

† Pearson Chi-squared test; ‡ Kruskal–Wallis H test; * *p* < 0.05 statistically significant; ** *p* < 0.01 highly statistically significant. **LEGEND**: **LMR** = agreement with the use of multimedia resources; **RMT** = agreement score for classic oral presentations; **RMP** = agreement score for PowerPoint presentations; **RMF** = agreement score for educational videos; **RMO** = agreement score for online documentary sources.

**Table 9:** Main features of the five-clusters solution.

	Final Cluster Centers—The Typical Case for Each Cluster					ANOVA		Distances Between Final Cluster Centers
	Cluster					F	*p*-Value	
	1 (n = 58)	2 (n = 46)	3 (n = 133)	4 (n = 151)	5 (n = 135)			
	Mean ± SD	Mean ± SD	Mean ± SD	Mean ± SD	Mean ± SD			
MPS	179.79 ± 8.311	109.59 ± 8.196	159.32 ± 4.951	142.78 ± 4.730	127.34 ± 4.262	1601.416	<0.001 **	1–2: 77.306
SAG	40.50 ± 2.637	28.67 ± 4.784	37.50 ± 3.368	34.66 ± 3.508	30.24 ± 3.028	157.543	<0.001 **	1–3: 22.627
LTG	35.00 ± 2.534	22.98 ± 3.343	31.46 ± 2.687	29.04 ± 3.282	25.78 ± 2.994	166.375	<0.001 **	1–4: 40.891
PDT	39.19 ± 3.600	19.35 ± 4.095	32.56 ± 4.281	27.68 ± 4.264	24.73 ± 3.322	234.065	<0.001 **	1–5: 57.731
FDT	35.29 ± 2.347	19.07 ± 3.349	30.99 ± 2.673	26.67 ± 2.751	23.70 ± 2.519	360.930	<0.001 **	2–3: 54.713
RUG	29.81 ± 4.375	19.52 ± 3.613	26.80 ± 4.367	24.73 ± 3.738	22.89 ± 3.171	63.777	<0.001 **	2–4: 36.454
								2–5: 19.681 3–4: 18.286 3–5: 35.182 4–5: 17.015

** *p* < 0.01 highly statistically significant. **LEGEND**: **MPS** = global indicator of motivational persistence; **SAG** = attitude toward setting up ambitious goals; **LTG** = attitude toward following goals in the long term; **PDT** = attitude toward planning daily tasks; **FDT** = attitude toward fulfilling daily tasks; **RUG** = attitude toward recalling unachieved goals.

**Table 10:** Additional features of the five-clusters solution.

		Cluster										*p*-Value
		1 (n = 58)		2 (n = 46)		3 (n = 133)		4 (n = 151)		5 (n = 135)		
		N	%	N	%	N	%	N	%	N	%	
Gender	male	8	13.8%	14	30.4%	32	24.1%	39	25.8%	32	23.7%	0.325 †
	female	50	86.2%	32	69.6%	101	75.9%	112	74.2%	103	76.3%	
Age group	18–20 years	37	63.8%	25	54.3%	77	57.9%	86	57.0%	72	53.3%	0.617 †
	21–24 years	11	19.0%	14	30.4%	38	28.6%	50	33.1%	44	32.6%	
	over 25 years	10	17.2%	7	15.2%	18	13.5%	15	9.9%	19	14.1%	
LMR	1	2	3.4%	1	2.2%	4	3.0%	7	4.6%	6	4.4%	0.002 †**
	2	4	6.9%	5	10.9%	14	10.5%	21	13.9%	34	25.2%	
	3	19	32.8%	21	45.7%	50	37.6%	59	39.1%	57	42.2%	
	4	11	19.0%	9	19.6%	32	24.1%	41	27.2%	18	13.3%	
	5	22	37.9%	10	21.7%	33	24.8%	23	15.2%	20	14.8%	
		m ± SD min ÷ max/median		m ± SD min ÷ max/median		m ± SD min ÷ max/median		m ± SD min ÷ max/median		m ± SD min ÷ max/median		
RMT		4.06 ± 1.026 1.06 ÷ 5.00/4.25		3.76 ± 0.839 2.06 ÷ 5.00/3.88		4.09 ± 0.976 1.00 ÷ 5.00/4.25		4.14 ± 0.815 1.06 ÷ 5.00/4.38		3.93 ± 0.881 1.50 ÷ 5.00/4.06		0.014 ‡*
RMP		3.81 ± 0.644 2.25 ÷ 5.00/3.97		3.04 ± 0.759 1.31 ÷ 4.69/2.94		3.59 ± 0.800 1.31 ÷ 5.00/3.69		3.37 ± 0.880 1.00 ÷ 5.00/3.50		3.31 ± 0.834 1.06 ÷ 5.00/3.31		<0.001 ‡**
RMF		3.96 ± 0.852 1.06 ÷ 5.00/4.06		3.31 ± 0.861 1.06 ÷ 4.69/3.25		3.74 ± 0.853 1.06 ÷ 5.00/3.94		3.64 ± 0.810 1.00 ÷ 5.00/3.81		3.33 ± 0.926 1.00 ÷ 5.00/3.56		<0.001 ‡**
RMO		3.49 ± 1.097 1.00 ÷ 5.00/3.81		3.06 ± 1.039 1.06 ÷ 4.94/3.03		3.51 ± 0.905 1.06 ÷ 5.00/3.50		3.26 ± 0.874 1.00 ÷ 5.00/3.25		3.15 ± 0.891 1.00 ÷ 5.00/3.13		0.002 ‡**

† Pearson Chi-squared test; ‡ Kruskal–Wallis H test; * *p* < 0.05 statistically significant; ** *p* < 0.01 highly statistically significant. LEGEND: LMR = agreement with the use of multimedia resources; RMT = agreement score for classic oral presentations; RMP = agreement score for PowerPoint presentations; RMF = agreement score for educational videos; RMO = agreement score for online documentary sources.

## Data Availability

The data presented in this study are available on request from the corresponding author. The data are not publicly available due to ethical and privacy restrictions.
